# Development and Validation of a Game-Based Assessment for Complex Problem Solving

**DOI:** 10.3390/jintelligence13010009

**Published:** 2025-01-14

**Authors:** Jian Li, Yi Ming Li, Yun-Xuan Xing, Bo Zhang, Yun Tang, Fritz Drasgow

**Affiliations:** 1 Faculty of Psychology, Beijing Normal University, Beijing 100875, China; jianli@bnu.edu.cn (J.L.); 202111061007@mail.bnu.edu.cn (Y.-X.X.); 2 Beijing Key Laboratory of Applied Experimental Psychology, Beijing 100875, China; 3 School of Ideological and Political Education and Moral Education, Beijing Institute of Education, Beijing 100120, China; liym@bjie.ac.cn; 4 School of Labor and Employment Relations, University of Illinois Urbana-Champaign, Champaign, IL 61820, USA; bozhang3@illinois.edu (B.Z.); fdrasgow@illinois.edu (F.D.); 5 Department of Psychology, University of Illinois Urbana-Champaign, Champaign, IL 61820, USA; 6 School of Psychology, Central China Normal University, Wuhan 430079, China; 7 Key Laboratory of Adolescent Cyberpsychology and Behavior, Ministry of Education, Wuhan 430079, China

**Keywords:** complex problem solving, planning–execution, metacognition, game-based assessment, achievement, Sokoban task, validation

## Abstract

Complex problem solving (CPS) refers to a set of higher-order capacities that allow an individual to interact with a dynamic environment and solve complex problems. The purpose of this study was to develop and validate Sokoban, a game-based assessment of the planning–execution stage of the CPS framework proposed by PISA 2012. The psychometric properties of this instrument were examined in a large sample of Chinese students (*n* = 1145) ranging from elementary to tertiary education. The results supported the two-faceted nature of Sokoban, as well as providing preliminary evidence about criterion-related and predictive validity for the planning–execution stages of complex problem solving. These empirical results lend support to the validity of this game-based assessment, as well as its practical implications in educational settings.

## 1. Introduction

With the rapid development of information technology, great changes in individuals’ work and lives have taken place, featuring complex problems (Griffin et al. 2012). The successful handling of the challenges of complex problems requires many higher-order thinking skills, at the heart of which is complex problem solving (Griffin et al. 2012). Complex problem solving (CPS) refers to problem solvers’ capacities to interact with a dynamic task environment by exploring and integrating information that is not evident but necessary for a successful solution (Buchner 1995; Dörner and Funke 2017). In recent decades, CPS has drawn considerable attention from researchers in a variety of domains. The empirical evidence has shown that CPS is essential for important life outcomes. After controlling for general ability, CPS has been found to predict academic achievement (Wüstenberg et al. 2012), work performance (Danner et al. 2011), and career advancement (Mainert et al. 2015).

### 1.1. Conceptual Framework for CPS

The significant role of CPS has attracted attention in the field of psychological and educational assessment. According to the Organization for Economic Cooperation and Development (OECD), CPS is considered to be a main component of 21st century skills (Binkley et al. 2012). Researchers have proposed various theoretical frameworks concerning CPS, with the most popular ones proposed by Greiff et al. (2015a) and the Program for International Student Assessment (PISA; OECD 2012). In the former one, emphasis was placed on the complexity of the problem, and the processes of CPS were divided into two parts: (1) knowledge acquisition and (2) knowledge application. The PISA 2012 framework, however, focuses more on general problem-solving processes. It approaches CPS processes as consisting of four stages (OECD 2010):

(1) Exploring and understanding: building mental representations from information gathered via observation, interaction, finding obstacles, and identifying relevant concepts;
(2) Representation and formulation: identifying relevant problem factors and cross-relationships for the purpose of constructing a coherent mental representation;
(3) Planning and execution: clarifying goals, devising strategies, and executing plans to achieve a goal state;
(4) Monitoring and reflection: checking progress at each stage, critically evaluating assumptions from different perspectives, and identifying needs for additional information.

According to the descriptions in PISA 2012, these stages are not necessarily sequential, nor are they required in all situations (OECD 2010). We believe it is important to measure the effects of the individual stages when assessing problem-solving performance, but few efforts have been made to examine these processes empirically (Sun et al. 2022). In this article, we specifically focus on the planning–execution stage of CPS, which includes both cognitive and metacognitive components. By introducing the Sokoban task, we measured participants’ planning and execution performance within the CPS framework through a game-based assessment (GBA).

### 1.2. Game-Based Assessment for CPS

Although PISA is a reliable tool for assessing problem-solving skills, it leans more towards evaluating general problem-solving performance rather than complex problem-solving ability specifically (see Sun et al. 2022 for a discussion). Furthermore, being a highly formal assessment tool, PISA is less accessible for practical applications in everyday life.

In contrast, game-based assessment (GBA) can effectively address these issues. GBA refers to a type of assessment that uses players’ interactions with the game as a source of evidence to make meaningful inferences about what players know and can do (i.e., knowledge, skills) and how individual players interact with the game as a problem-solving process (Gomez et al. 2022). GBA has great potential in assessing constructs that are difficult to measure by traditional paper-and-pencil tasks (Buckley et al. 2021; Das and Misra 2014; Drasgow 2015; Georgiou and Das 2016; Georgiou et al. 2017; Goslen et al. 2024; Mislevy et al. 2015; Wang et al. 2012). Given that GBA typically features contexts without fixed learning goals or methods, where problem-solving processes are shaped by individuals’ self-regulated learning abilities, it accurately represents the circumstances when tackling complex problems in daily life (Sun et al. 2022). Especially for higher-order constructs like CPS, it shows promise for improved construct-related validity (Griffin et al. 2012).

In the 1970s, a number of computer-simulated microworlds were developed to resemble real-life situations to improve the generalizability of empirical findings (Frensch and Funke 1995). Nonetheless, measurement issues, such as low reliability and the violation of local independence, overshadowed the advantages (Wüstenberg et al. 2012). Later, two formal models—Linear Structural Equation systems (LSEs; Funke 1993) and Finite State Automata (FSAs; Buchner and Funke 1993)—were introduced to describe the structure of a dynamic task independent of its semantic content (Greiff et al. 2013a). An LSE is a mathematical model capable of illustrating the linear relationships between various variables and their dynamic changes. This model facilitates the analysis and comprehension of systemic behavioral patterns (Funke 1993). In contrast, FSA is rooted in computer science, describing the different states a system might be in and the transition rules between these states on an event-driven basis. The advantage of FSA lies in its ability to effectively capture the logical structure of decision making and feedback within problem-solving processes (Buchner and Funke 1993).

The LSE-based MicroDYN and GeneticsLab and the FSA-based MicroFIN were developed to overcome the psychometric shortcomings of early microworlds (see Greiff et al. 2013a for more details), and several studies provided evidence in support of their psychometric value (e.g., Greiff et al. 2013a, 2015b; Schweizer et al. 2013; Wüstenberg et al. 2012).

Notwithstanding the popularity of these tasks, they have some important limitations. First, the tasks were developed under minimal complex systems (Greiff et al. 2012) and thus were relatively simple (Funke 2014). Second, the scenarios used in the LSE-based MicroDYN and GeneticsLab were very homogenous because they involved a deterministic causal analysis. For the most part, a single strategy called VOTAT (varying one thing at a time) was needed to solve all the problems and could account for a large portion of the overall performance variance (see Funke 2014; Greiff et al. 2015b; Wüstenberg et al. 2012). Homogenous scenarios and heavy reliance on a single strategy may severely narrow the construct being measured (Neubert et al. 2014). Furthermore, these tasks relied on outcome-oriented indicators (i.e., whether the problem can be solved or not). As CPS is essentially dynamic, process-oriented measures can provide an incremental value in predictive validity (Greiff et al. 2013a). Thus, a combination of process-oriented and outcome-oriented indicators is needed to better capture the nature of CPS.

### 1.3. Cognitive and Metacognitive Components in CPS

To reliably and accurately assess CPS, it is important to understand its underlying mechanisms. Early assessment practices treated CPS as a unidimensional construct and neglected the development of a formal framework to account for CPS (see Funke 2012). To derive a theoretical framework, researchers may gain some insights from theories of general problem solving. For example, the IDEAL model (Bransford and Stein 1984) conceptualized problem solving as a unified process consisting of five phases. Similarly, Sternberg (1986) proposed a model to describe seven stages that problem solvers typically go through. Both models recognized several aspects in problem solving, including the identification and representation of the problem, the exploration and development of strategies, and the evaluation of the solution. From a process-oriented perspective, all of these theories of problem solving emphasized metacognitive and cognitive components.

Metacognition refers to higher-order thinking in which people actively control the cognitive processes involved in learning and problem solving (Davidson et al. 1994). Empirically, the inclusion of metacognition in CPS is also likely to improve the predictive value of CPS. Metacognition has shown predictive validity for inductive learning over and above the effects of cognitive ability (Veenman et al. 2006) and in work-related contexts (Das and Misra 2014; Keith and Frese 2005; Schmidt and Ford 2003; Sitzman and Ely 2011).

The inclusion of metacognition can be especially useful for understanding CPS (Funke 2010). For example, the PISA 2012 framework proposed individual stages that included both metacognitive and cognitive components (OECD 2010). Specifically, planning and execution were involved in the problem-solving processes, which required a metacognitive (setting goals and adapting them dynamically) and cognitive (reasoning and executing plans) effort, respectively. Some studies also investigated how metacognition was involved in CPS (Greiff et al. 2016; Güss et al. 2010), although they did not focus on the assessment of metacognition as a component of CPS.

Inspired by PISA 2012, we propose and test here a two-faceted CPS framework consisting of planning and execution. Planning refers to the metacognitive processes through which an individual recognizes, defines, and represents a problem, sets goals, and acquires the knowledge necessary for the solution. Execution includes the cognitive processes that develop and implement solutions by applying the information carried on from planning and modify this information if necessary. These two components are empirically separable and target different phases in solving complex problems. Using this framework, we aim to emphasize the importance of metacognition and cognition, introducing a combination of process-oriented and outcome-oriented measures in the assessment of CPS.

To add to the current literature, the present study focused on the planning–execution stage of CPS and developed a game-based assessment based on this framework. We conducted three studies to evaluate the psychometric properties of this CPS measure.

## 2. Study 1

In Study 1, we sought to examine the factor structure of CPS with the use of a game-based assessment, namely the Sokoban task (Jarušek and Pelánek 2010). Since our measure of CPS was based on the planning–execution framework, we hypothesized that a two-factor latent structure would provide an adequate fit to the Exploratory Factor Analysis and Confirmatory Factor Analysis results.

### 2.1. Method

#### 2.1.1. Participants

The participants of Study 1 included 981 students from eight schools and five universities in China of different levels. More specifically, we recruited students from elementary (5th and 6th grade), junior (7th and 8th grade), and senior (10th and 11th grade) schools and college using a cluster sampling method. Table 1 presents information on the sample size, age, gender, and school level for the whole sample. Ethics permission was obtained from the Ethics Committee of the Faculty of Psychology, Beijing Normal University for studies involving humans (BNU202403070058). For our child participants, we also obtained parental consent prior to testing.

#### 2.1.2. Measures

##### Sokoban Task

A complex problem has five major characteristics: the complexity of the structure, the interconnectedness of the variables, the polytely (multiple goals) of the task, the nontransparency of the situation, and the dynamics of the system (Funke 2012). According to this, we believe that the game Sokoban can be a good candidate to assess complex problem solving.

**Figure 1:**
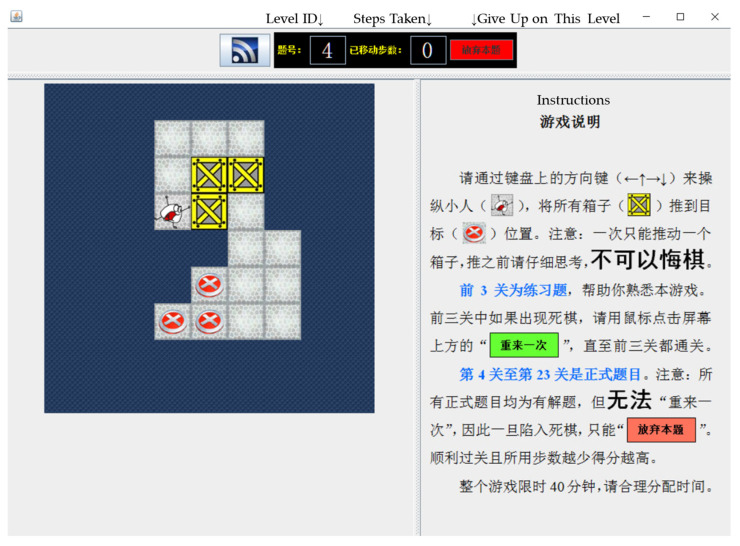
A screenshot of a Sokoban task. Note. The general meaning of the instructions in Figure 1 is as follows: Use the arrow keys to control the character and push boxes to their target positions. One box at a time, with no withdrawal. The first 3 levels are practice sessions where you can click “Restart” to reconsider your choice. Levels 4 to 23 are formal challenges with no “Restart” option; If you reach a deadlock, the only option is to “Abandon this level”. Fewer moves and less time result in higher scores. The game is limited to 40 min, so plan your time wisely.

Sokoban is a classic Japanese puzzle game (see Figure 1) composed of a pusher (the white character in the lower right corner), *n* boxes (*n* ≥ 1, indicated by the yellow cells), and *n* storage locations (indicated by the red plates). Participants were asked to push the yellow boxes into the storage in the minimum number of moves. They needed to control the pusher to push boxes by using the arrow keys on the keyboard. The task contained three practice scenarios and twenty test scenarios. Instructions were always presented on the right side of the screen. The practice sessions had an unlimited number of attempts for participants to get familiar with the task. Participants had to successfully solve the three scenarios before starting the test session. In the test session, participants were informed that withdrawal was not allowed. There was no time limit, but only one attempt was allowed for each scenario. The test scenarios were arranged by ascending difficulty based on data from a pilot study (reported in the Supplementary Materials).

We chose Sokoban for the following characteristics, which highly align with CPS: (1) different scenarios require diverse strategies to solve them; (2) frequent consideration of the interactions between the pusher and the boxes is required; (3) which target location sa certain box should be placed in is unknown; (4) each scenario cannot be completed following a simple rule; (5) the scenarios cover a wide range of difficulty levels; and (6) it is interesting enough to motivate participants, as well as having a low requirement of special knowledge.

For each scenario, we derived an index of planning and an index of execution. Inspired by previous index settings for the problem-solving task (Wang et al. 2023), the index of planning was calculated as the ratio between planning time (the time elapsed between the presentation of the scenario and the first push) and total time (the time elapsed between the presentation of the scenario and the completion of the scenario). It is a continuous variable ranging from 0 to 1. Higher scores indicate better planning (Li et al. 2015). The index of execution was measured by the solution status, namely, whether each scenario was successfully solved. It is a dichotomous variable. The scores for the planning and execution indexes across the 20 scenarios were averaged to calculate two overall indexes, which ranged from 0 to 1.

#### 2.1.3. Procedure

The elementary, junior, and senior students completed the Sokoban test in groups using computer classrooms in their respective school. One or two trained experimenters were in charge of the test administration, while the head teacher of each class was present to keep discipline during the test. The college students completed the Sokoban task individually in a psychology lab at Beijing Normal University. Testing lasted approximately 30 min for the college students and 40 min for the others.

#### 2.1.4. Data Analysis

An Exploratory Factor Analysis (EFA) and Confirmatory Factor Analysis (CFA) were conducted to examine the structural validity of Sokoban for the planning–execution stage of CPS. The CFA was performed in Mplus 7.0 (Muthén and Muthén 2012). Table S1 in the Supplementary Materials presents additional technical details of measurement invariance tests across gender and cohort groups according to Chen (2007).

### 2.2. Results and Discussion

Table 2 shows the Cronbach’s α, mean, and standard deviation for the total accuracy of execution and planning in each cohort sample. The Cronbach’s αs range from 0.79 to 0.87 for planning and from 0.77 to 0.83 for execution, suggesting a good internal consistency.

To test the factorial structure of the planning–execution stage, we first conducted an EFA with half of the data. Principal axis factoring was chosen as the extraction method, and promax rotation was applied to the factor solution. The scree plot revealed a distinct break after the second factor, with a sharp decline in the eigenvalues for subsequent factors, which supported the retention of two factors. Following the results of the EFA, we fixed the factor number as two. Further results showed that a two-factor model performed well. The two factors could be interpreted as execution, with factor loadings ranging from 0.30 to 0.66, and planning, with factor loadings ranging from 0.33 to 0.62, respectively. The correlation between planning and execution was *r* = 0.66, suggesting that planning and execution were two correlated but still distinct factors. The CFA was then conducted with the other half of the data to provide more evidence in support of the planning–execution CPS framework. The results indicated that the two-factor model fitted the data well (χ2 = 1212.782, df = 719, χ2/df = 1.69, CFI = 0.93, TLI = 0.93, RMSEA = 0.03, SRMR = 0.05). The standardized factor loadings for planning ranged from 0.43 to 0.62, and they ranged from 0.36 to 0.66 for the execution factor.

In addition, since the two components of CPS are expected to improve with the progression of adolescence (OECD 2010), an ANOVA was conducted to test the effect of age (elementary, junior, senior, and college) on both planning and execution scores. The results showed a significant effect of age on planning scores, *F*(3, 977) = 108.50, *p* < .001, as well as on execution scores, *F*(3, 977) = 254.39, *p* < .001. Post hoc comparisons using Scheffe’s method indicated that the differences in planning and execution scores between all age groups were statistically significant. Thus, both components showed a gradual improvement as age increased, providing evidence for the construct validity of the CPS measure.

Taken together, these findings about the planning–execution structure provided support of the two-faceted nature of CPS, and they were in line with the results in previous studies (e.g., Graesser et al. 2017; Li et al. 2020). Moreover, the age-related results provided preliminary evidence for the construct validity of the measure.

## 3. Study 2

Having established the two-factor structure of Sokoban, it is also important to provide criterion-related validity evidence for these stages. Thus, in Study 2, we sought to examine how well the planning and execution scores of Sokoban relate to an established measure of CPS and to other cognitive processing skills. As a widespread and established measure for domain-general CPS, GeneticsLab (Sonnleitner et al. 2012) was used as the criterion to validate Sokoban as an effective measure for assessing CPS. It was also noticed that the whole process of solving a complex problem placed demands on working memory capacity (Greiff et al. 2015c; Schweizer et al. 2013; Wüstenberg et al. 2012) and fluid reasoning ability (Wüstenberg et al. 2016). Specifically, in the planning processes, participants have to encode and store relevant information, mentally manipulate relevant information, and update stored information after manipulation, which requires working memory capacity (e.g., Wiley and Jarosz 2012). On the other hand, participants only need to use information to work towards the solution of a problem during the execution phase, which resembles common reasoning tasks (e.g., Kretzschmar et al. 2016).

Based on this, we hypothesized that the planning and execution scores of the Sokoban task would be positively related to GeneticsLab. Moreover, we hypothesized that the score in the planning component of Sokoban would correlate significantly with working memory measured by Digit–Letter Sequence and Digit Span, and the score in the execution component of Sokoban would correlate significantly with reasoning as measured by the Raven Advanced Progressive Matrix.

### 3.1. Method

#### 3.1.1. Participants

Study 2 was conducted with college students, with the samples for RAPM, GL, and DLS/DS mutually exclusive. The numbers of participants of RAPM, GL, and DLS/DS were 36, 106, and 82, and their average age (SD) was 21.61 years (2.20), 19.63 years (1.41), and 20.61 years (2.18), respectively.

#### 3.1.2. Measures

##### Sokoban Task

We administered the same measure described in Study 1.

##### GeneticsLab

Rooted in the MicroDYN approach, GeneticsLab (GL) is an established measure for domain-general complex problem solving, which contains two processes named knowledge acquisition and knowledge application (Greiff et al. 2015a). Participants first explored how the physical features (e.g., height or weight) of a fictional creature were influenced by certain genes and recorded the connections. Then they were asked to create a creature with certain features within a limited number of gene manipulations. Twelve validated scenarios were used. The scores ranged from −0.5 to 0.56 for knowledge acquisition and from 0 to 1 for knowledge application. No time limit was imposed on practice scenarios, while 35 min was allowed for test sessions. The Cronbach’s α for knowledge acquisition and knowledge application were 0.86 and 0.85, respectively.

##### Digit–Letter Sequence and Digit Span

Digit–Letter Sequence (DLS) is a working memory capacity subtest in the Wechsler Adult Intelligence Scale–Third Edition (WAIS-III; Wechsler 1997). During the test, the test administrator read a sequence of numbers and letters to the participant. The participant was required to recall all the numbers in ascending order and all the letters in alphabetic order. The length of the sequence increased as the test became more difficult. For each length, there were three sequences. When the participant failed all three sequences of the same length, the test stopped. The maximum score of this test is 21. No time limit was imposed.

Digit Span (DS) is a working memory subtest of the WAIS-III. It consists of a forward span and backward span. The test administrator read a sequence of numbers to the participant. In the forward span test, the participant was required to rehearse exactly what he heard. In the backward span test, the participant was expected to rehearse what he had heard in reverse order. The length of the sequence increased as the test became more difficult. For each length, there were two sequences. When the participant failed both sequences, the test was discontinued. The maximum score was 20 for the forward span test and 18 for the backward span. No time limit was imposed.

##### Raven Advanced Progressive Matrix

The Raven Advanced Progressive Matrix (RAPM) was used to assess fluid reasoning (Raven and Court 1998). There were 36 items, and the maximum score was 36. Each item consisted of a 3 × 3 matrix, of which the right lower element was missing. Participants needed to determine how the elements change and select the correct element from eight options to complete the matrix. Thirty minutes was allowed for the test. The Cronbach’s α was 0.87.

#### 3.1.3. Procedure

The RAPM was administered in a group setting in a quiet conference room, GL was administered in group in a computer classroom, and DLS/DS was administered individually by a trained experimenter in a psychology lab at Beijing Normal University. All the students completed the same Sokoban task as in Study 1.

### 3.2. Results and Discussion

We assessed the criterion-related validity using sum score-based correlations between the game-based Sokoban measures and the criterion variables. The estimated correlation coefficients are presented in Table 3.

The results showed that knowledge application was positively correlated with planning (*r_app-pl_* = 0.32, *p* = .001) and execution (*r_app-ex_* = 0.30, *p* = .002). However, the correlations between knowledge acquisition and the two CPS components were not significant.

To examine the relationships between the CPS components and working memory capacity, a set of correlation analyses were performed. The results showed that the working memory score, which was the sum of the DS score and the DLS score, was significantly correlated with planning (*r_wm-pl_* = 0.25, *p* = .028) and execution (*r_wm-ex_* = 0.25, *p* = .027). Further investigation using the scores from the two working memory tasks separately revealed that the DS score was significantly correlated with execution (*r_ds-ex_* = 0.24, *p* = .034) but not with planning (*r_ds-pl_* = 0.20, *p* = .077). In turn, DLS, which was more difficult and needed more metacognitive strategies than DS, was significantly correlated with planning (*r_dls-pl_* = 0.25, *p* = .026) but not with execution (*r_dls-ex_* = 0.18, *p* = .120).

The RAPM is considered a representative measure of fluid reasoning (Carpenter et al. 1990). We found that the score in fluid reasoning was significantly correlated with execution but not with planning (*r_ra-ex_* = 0.47, *p* = .004; *r_ra-pl_* = 0.26, *p* = .128). Given the relatively large correlation coefficient between the Raven and planning, the nonsignificant result may be due to the small sample size. However, the pattern of results still suggested that fluid reasoning was more closely associated with execution than planning.

Overall, the combined results supported our hypothesis regarding criterion-related validity. Although the results showed that both planning and execution were significantly correlated with working memory, there was still some evidence indicating that the two components were distinct (this finding is addressed further in the general discussion), thus providing an acceptable criterion-related validity for the planning–execution framework.

## 4. Study 3

Study 1 examined the structural validity of the planning–execution framework, confirming a two-factor structure for CPS, while Study 2 explored the correlations between the GBA scores and cognitive abilities theoretically related to CPS. However, an unresolved question is whether GBA scores can predict students’ performance in real-world practice. Thus, the purpose of this study was to examine the predictive validity of the CPS measure. Previous studies have shown that both the planning and the execution components of CPS are significantly related to academic achievement (Greiff et al. 2014, 2013b; see also Cai et al. 2016; Das and Georgiou 2016; Sergiou et al. 2023). Since CPS has unique components beyond reasoning ability (Wüstenberg et al. 2012), it should account for the extra variance in academic achievement after controlling for reasoning. Moreover, since we expected a significant positive correlation between execution and reasoning, we further hypothesized that the scores in the planning index of the Sokoban task would account for a significant amount of variance in academic achievement.

### 4.1. Method

#### 4.1.1. Participants

The validation sample consisted of students from a junior (7th and 8th grade) school and a senior (10th grade) school in China. The numbers of participants in each grade were 126 (7th), 160 (8th), and 107 (10th), and their average age (*SD*) was 12.62 years (0.40), 13.70 years (0.47), and 15.69 years (0.33), respectively. Parental consent was obtained prior to testing.

#### 4.1.2. Measures

##### Sokoban Task

We administered the same task as the one described in Study 1.

##### Academic Achievement

Following the common course design in secondary education in China, AA was assessed by Chinese, Math, and English scores for the 7th grade students; by Chinese, English, History, Math, Physics, and Biology for the 8th grade students; and by Chinese, English, History, Math, Physics, and Chemistry for the 10th grade students. Different subjects are used to calculate AA for different grades because only these subjects have formal exams in the semesters of Grades 7, 8, and 10, respectively. Thus, these subjects reflected the academic requirements for the corresponding grade level. The raw scores were converted into standard scores based on the grade mean and standard deviation, and then the average score for each individual was calculated as AA.

#### 4.1.3. Procedure

To operationalize academic achievement, we accessed and used the final exam scores of each student after they had finished the Sokoban task in the semester in which the study was conducted. Ethics permission was obtained from the Ethics Committee of Faculty of Psychology, Beijing Normal University (Approval number 202403070058), and we also obtained parental consent prior to testing children in Sokoban.

### 4.2. Results and Discussion

We employed a model comparison approach to examine the predictive validity of planning and execution. Two sets of linear regression analyses were conducted in each cohort sample to predict academic performance. Since reasoning is consistently found to be a significant predictor of academic achievement (Georgiou et al. 2020; Neisser et al. 1996) and gender differences in academic achievement are also well documented (Marsh and Yeung 1998; Torppa et al. 2018), we used gender and reasoning as control variables in Model 1 (the baseline model). Model 2 included gender, reasoning, and the two CPS components as the predictors. The improved model fit of Model 2 over Model 1 shows the unique contribution of CPS after controlling for reasoning and gender, which provides evidence for predictive validity. Information about model fit and the coefficients can be found in Table 4. For Grade 7 and Grade 10, CPS showed no incremental value in accounting for academic achievement. For Grade 8, CPS explained a significant part of the variance in academic achievement (Δ*R*^2^ = 0.05, *p* = .005). A further inspection of the regression coefficients suggested the different roles of execution and planning, as expected. Planning exhibited a significant effect on the overall academic performance in Grade 8 and Grade 10. In contrast, execution showed no effect on academic achievement.

These results suggest that planning and execution are separate constructs that may have a difference influence on academic achievement, providing further support for the two-factor model of CPS.

## 5. General Discussion

There is a growing recognition that CPS is important in real life. But the theoretical basis needs further investigation, and valid instruments are still in great need. Therefore, the present study proposed that one stage of CPS can be conceptualized as a two-faceted construct consisting of planning and execution, and we developed a new measure based on this framework. A significant theoretical contribution is made by explicitly incorporating a metacognitive component into the CPS theoretical framework, and an empirical contribution is made by developing the Sokoban task as a new measure of CPS. The present study also represents our attempt to keep abreast with GBA. We carefully designed reliability and validity studies and examined the psychometric properties in multiple samples. The results of this study provide preliminary evidence in support of the reliability and validity of Sokoban as a measure of CPS.

### 5.1. The Validity of the Game-Based Assessment of CPS

In the present study, we evaluated the validity of the Sokoban task from three aspects: the structural validity from CFA and EFA results, the criterion-related validity between CPS and other measures, and the predictive validity.

First, an EFA was conducted to test the factorial structure of the planning–execution stage in CPS, and the results showed that a two-factor structure performed well. CFA results provided further evidence supporting the planning–execution model. Planning and execution were moderately correlated, demonstrating that CPS can be conceptualized as two correlated but distinct factors.

Second, the relationship between Sokoban performance and other measures suggested an acceptable criterion-related validity. Knowledge application in GL was significantly correlated with the two CPS components, whereas knowledge acquisition was not. This is probably because we focused on the third stage of CPS as outlined in the PISA framework, whereas “knowledge acquisition” should be more closely related to the first two stages, theoretically. Moreover, the significant positive correlation between planning and working memory, as well as the significant positive correlation between execution and reasoning, validates our hypothesis regarding criterion-related validity. However, the result showed that execution was significantly correlated with working memory, which is not what we expected to find. There might be a reason for this result. During the execution phase, individuals must keep the designed path in mind long enough for it to guide one’s action and to execute selected strategies, which also required working memory capacity (Imbo et al. 2007; Spiegel et al. 2013; Zelazo et al. 1997). This does not imply that the two stages are indistinguishable or difficult to differentiate, but rather that both stages require the heterogeneous application of working memory.

Third, we examined the predictive validity of CPS by controlling for the effect of reasoning and gender when predicting academic performance. Overall, the results were somewhat mixed. When predicting academic achievement in Grade 8, CPS measures accounted for 5% of the unique variance in academic achievement beyond gender and reasoning, which resembled the results in MicroDYN (Greiff et al. 2015c; Greiff and Neubert 2014; Schweizer et al. 2013). However, the same pattern was not observed in the 7th and 9th grades. It should be mentioned here that the academic achievement score in the different grade levels represented performance in different subject areas, and this may have attenuated the correlations. Keeping in mind that different components of planning may predict different academic outcomes (e.g., Das and Georgiou 2016), using an outcome measure that comprised different subject areas may have impacted our results.

### 5.2. The Planning–Execution Framework

Most researchers have studied problem solving from an information-processing perspective, despite the fact that opinions about the specific phases differ (e.g., Bransford and Stein 1984; Das and Misra 2014; Novic and Bassok 2005; Sternberg 1986). The present study followed this tradition by proposing a planning–execution framework of CPS. The information-processing view is beneficial for understanding what happens in the brain when participants attempt to solve a complex problem. In this study, metacognition—planning, specifically—was explicitly proposed as a critical phase of complex problem solving, in which problem solvers set goals and built mental models of a problem. In the execution phase, cognitive abilities such as reasoning and working memory operate on these mental models towards the goal. In the resulting planning–execution framework, metacognition and cognition are successfully unified but are still separable.

It may be argued that planning and execution are not independent, since the two components seems to be highly correlated. However, we believe that they are distinct factors in at least two ways. Theoretically, planning is a metacognitive process where an individual needs to understand the problem, set goals, and acquire the necessary knowledge to solve it. In contrast, execution is a cognitive process where the individual applies knowledge in the process of problem solving. Empirically, the execution score was significantly correlated with DS, whereas the planning score was correlated with DLS, which was more difficult and needed more metacognitive strategies than DS, underscoring that they are distinct components. In sum, the planning–execution framework distinguishes between two components within an individual stage of CPS, offering some new insights into understanding the nature of CPS.

### 5.3. Limitations and Future Research

The present study has some limitations worth reporting. First, the restriction of move revocation may amplify the measurement error, because it is common to observe that inadvertent moves lead to accidental failures. Nevertheless, participants were given enough time in the present study, which should have alleviated this problem. Second, the findings about criterion-related validity are somewhat preliminary; since we only measured the planning–execution stage instead of the whole process of CPS, there is a relatively low correlation between the index of planning–execution and other CPS measures (e.g., Sonnleitner et al. 2012). Future studies aiming to measure a specific stage within CPS should also be aware of this issue and select more appropriate criterion tasks tailored to those stages. Third, while our study provides initial insights into the predictive validity of Sokoban, the results are mixed and warrant further investigation. Future research should focus on examining the predictive validity of Sokoban across diverse contexts to better understand its applicability. Fourth, due to the limited sample sizes for each age group in the present study, we were unable to conduct an age-specific data analysis. Future studies could include larger samples and use structural equation modeling for multiple comparisons to examine whether the measurement models are equivalent across different age groups. Fifth, in predicting academic achievement, we only measured the planning–execution stage in CPS using the Sokoban task, which may cause methodological artifacts. Future studies should use multiple measures to draw a more valid construct-level conclusion. Last but not least, CPS is an important construct not only in academic achievement but also in many other areas in education or related fields. Future studies should expand the scope and explore the role of CPS in other educational or workplace outcomes.

### 5.4. Implications

Using Sokoban as a game-based assessment of CPS has some practical implications. First, it is easy to generate strictly parallel items of Sokoban. Every original item and its mirror image can be rotated 90°, 180°, and 270°. These rotated items are strict isomorphs of the original one (Faber et al. 2009) but are hard to recognize, making them good candidates for parallel tests. Second, compared with VOTAT tasks, Sokoban items are heterogeneous and require complex strategies and operations. This feature also makes Sokoban less susceptible to practice effect. Third, the Sokoban measures are derived based on the underlying planning–execution framework of CPS. Therefore, we can determine the phases in which a problem solver shows weaknesses and initiate targeted intervention programs. These features render Sokoban especially valuable in contexts like clinical and educational intervention studies and personnel selection (see Das and Misra 2014 for a discussion of this).

## 6. Conclusions

The present study incorporated metacognitive and cognitive components into a unified CPS framework based on PISA 2012, developed Sokoban (a game-based assessment), and provided support for its psychometric properties. The results testified to the two-faceted structure of planning–execution and offered initial evidence for the criterion-related validity of Sokoban measures, as well as their predictive validity in predicting academic performance CPS. Therefore, we conclude here that Sokoban is a valid assessment of CPS and can be applied in educational settings.

## Figures and Tables

**Table 1:** Demographical information of participants in Study 1.

	Elementary		Junior		Senior		College	
	M	F	M	F	M	F	M	F
*N*	96	86	149	138	91	88	97	236
Age SD	11.28 0.68	11.20 0.68	13.30 0.74	13.15 0.65	16.40 0.93	16.22 0.86	21.15 2.15	20.28 1.23

**Table 2:** Descriptive statistics and Cronbach’s α in each cohort sample.

Cohort	*N*	Planning		Execution	
		α	*M* (*SD*)	α	*M* (*SD*)
Elementary	182	0.80	0.45 (0.09)	0.80	0.34 (0.20)
Junior	287	0.85	0.48 (0.11)	0.82	0.50 (0.23)
Senior	179	0.79	0.54 (0.10)	0.83	0.70 (0.21)
College	333	0.85	0.59 (0.10)	0.77	0.80 (0.17)

**Table 3:** Descriptive statistics of criterion variables and correlations with CPS measures.

Criterion	Descriptive Statistics		Sum Score Correlation	
	*n*	*M*(*SD*)	Planning	Execution
GL.acq	106	0.45 (0.13)	0.15	0.10
GL.app	106	0.80 (0.18)	0.32 **	0.30 **
WM	82	44.56 (6.85)	0.25 *	0.25 *
DS	82	30.34 (5.09)	0.20	0.24 *
DLS	82	14.04 (2.91)	0.25 *	0.18
Raven	36	26.47 (4.02)	0.26	0.47 **

Note. GL.acq = GeneticsLab knowledge acquisition, GL.app = GeneticsLab knowledge application, WM = working memory, DS = Digit Span, DLS = Digit–Letter Sequence. * *p* < .05, ** *p* < .01.

**Table 4:** Results of regression analysis predicting academic achievement.

	Grade 7		Grade 8		Grade 10	
Predictors	Model 1	Model 2	Model 1	Model 2	Model 1	Model 2
Gender	−0.22 **	−0.21 *	−0.20 **	−0.20 **	0.07	0.06
Reasoning	0.47 ***	0.48 ***	0.39 ***	0.36 ***	0.28 **	0.26 **
Execution		−0.03		0.02		−0.04
Planning		0.07		0.22 *		0.18 *
*R* ^2^	0.30	0.30	0.20	0.25	0.08	0.11
Δ*R*^2^		0.00		0.05 **		0.03

Note. * *p* < .05, ** *p* < .01, *** *p* < .001.

## Data Availability

The data presented in this study are available on request from the corresponding author.

## Supplementary Materials

The following supporting information can be downloaded at: https://www.mdpi.com/article/10.3390/jintelligence13010009/s1, Table S1. Model comparison results for measurement invariance across gender and cohort groups.
