# Supplementary material for: Development and Validation of a Game-Based Assessment for Complex Problem Solving
Source: J Intell. 2025 Jan 14;13(1):9. doi: 10.3390/jintelligence13010009 (PMC11765979; doi:10.3390/jintelligence13010009)
Supplement: Supplementary file 1 [file jintelligence-13-00009-s001.zip › jintelligence-3351344-supplementary.pdf]

1. Test development of Sokoban

Task selection. Because CPS emphasizes interactions between the problem solver and the problem context, a computer-based task is critical for effective assessment (Schweizer, Wüstenberg, & Greiff, 2013). Sokoban was selected and standardized to assess complex problem solving. Sokoban is a classic Japanese puzzle game composed of a pusher, n boxes ($n \geq 1$) and n storage locations.

We chose Sokoban for the following characteristics: (1) it is interesting enough to motivate participants; (2) it has a low requirement of special knowledge; (3) different scenarios require diverse strategies to solve; (4) each scenario can be completed within a relatively short time; and (5) the scenarios cover a wide range of difficulty levels. In this game, players need to control the pusher to push boxes by using the arrow keys on the keyboard. Players can push (cannot pull) only one box a time and a storage location can hold only one box. Sometimes, participants have to move away from the target state to some degree in order to push all boxes into the locations. Participants may also need to move multiple boxes alternatively when moving towards the target state. These strategies may be needed in one scenario. A scenario is considered successfully completed when all boxes are in storage locations. The problem space changes after every move, satisfying the dynamic feature of a complex problem. In some cases, the storage location for a given box is flexible. In some other cases, every box has to be pushed into a specific storage location in a specific order. Participants do not know whether the solution is flexible or fixed in the beginning, thus rendering the goal nontransparent. The interestingness and complexity of Sokoban were confirmed by Jarušek and Pelánek (2010) and Li, Zhang, Du, Zhu, and Li (2015).

Scenario development and indicator selection. Since multi-item testing is a necessary prerequisite for valid assessment (Greiff, Wüstenberg, & Funke, 2012), we first needed to develop multiple scenarios. Three rules were followed during scenario development: (1) only one chance was allowed for each scenario, (2) the first move should be essential for successful solution, and (3) withdrawal of moves was not allowed.

The following variables were recorded in each scenario: the time lapse between the presentation of the scenario and the first move (planning time), the time lapse between the presentation of the scenario and the completion of the scenario (total time), and whether the scenario is solved (solution status). For each scenario, we derived a planning indicator and an execution indicator. Specifically, the ratio between planning time and total time was used as the indicator of planning. This indicator is continuous ranging from 0 to 1. Higher score implies better planning. The time ratio has been proved a valid measure of planning (Li et al., 2015). The solution status, namely whether each scenario was successfully solved, was used as the indicator of execution. This indicator is dichotomous.

Twenty items were arranged by ascending difficulty based on pilot study data. Instructions were always presented on the right side of the screen. At first, participants filled in demographical information. Then they were administered three practice scenarios to familiarize them with the game, with an unlimited number of attempts. The three practice scenarios had to be successfully solved before they moved to the test session. In the test session, however, participants were not allowed to revoke moves and only one attempt was allowed for each scenario. The sum scores across the twenty scenarios were calculated. The maximum scores for planning and execution were both 20. No time limit was imposed.

2. Measurement invariance

Considering the diversity of our sample, we examined the measurement invariance of the CPS scores to see whether the latent structure held consistent across the sample. Specifically, we tested models of configural invariance, factor loading invariance, intercept invariance, and residual invariance. Since the chi-square test is very sensitive to sample size, even subtle differences will be statistically significant in large samples. Moreover, invariance testing involves a series of sequential tests, which can inflate the Type I error rate. Therefore, we considered chi-square test results, ΔCFA, and ΔRMSEA together when evaluating the final results. According to Chen (2007), when ΔCFA ≥ -.010 and ΔRMSEA ≤ .015 in large samples ($n > 300$), the invariance hypothesis can be accepted.

Table S1. Model comparison results for measurement invariance across gender and cohort groups.

Comparisons	Model	χ^2	df	CFI	RMSEA	$\Delta\chi^2$	Δdf	p	ΔCFI	ΔRMSEA
Male vs Female	Model 1	2351.309	1478	.942	.033					
	Model 2	1902.540	1515	.974	.022	18.187	37	.996	.032	-.011
	Model 3	1923.829	1534	.974	.022	26.304	19	.121	.000	.000
	Model 4	1925.157	1574	.977	.020	44.317	40	.295	.003	-.002
Cohort Groups	Model 1	3610.836	2956	.908	.031					
	Model 2	3661.579	3067	.916	.029	148.271	111	.011	.008	-.002
	Model 3	3753.102	3124	.911	.030	164.641	57	<.001	-.004	.001
	Model 4	3848.780	3244	.915	.029	153.691	120	.021	.004	-.001

Note. Model 1: Configural invariance; Model 2: Loadings and threshold invariance; Model 3: Loadings, threshold and intercept invariance; Model 4: Loadings, threshold, intercept, and residual invariance

In Model 1 (configural invariance), only factor loading patterns were constrained to be the same. In Model 2, we constrained all factor loadings and the thresholds of execution to be the same. In Model 3, we further constrained the intercepts of planning to be the same. In Model 4, item residuals were constrained to be the same. In implementing the models, we

followed Muthén and Muthén's (2012) suggestions for measurement invariance tests involving categorical indicators. Fit information and model comparison results are displayed in Table S1.

Male versus Female. The measurement invariance between male and female students was first examined. The satisfactory fit of Model 1 suggested that the two-factor structure held in both samples. Comparison between Model 2 and Model 1 implied that the factor loadings and the thresholds of execution were invariant across gender groups. Whereas comparison between Model 3 and Model 2 showed that the intercept invariance was held. Finally, comparison between Model 4 and Model 3 provided evidence for the invariance of residuals. In sum, measures derived from the CPS task was strictly invariant across gender.

Across cohort groups. We then examined whether the CPS measures were invariant across cohort groups (i.e., elementary, junior high, senior high, and college students). Similarly, we considered the goodness-of-fit of Model 1 and the subsequent model comparison results. Fit of Model 1 suggested a good model fit of the two-factor structural. Taking the large sample size ($n > 1000$) into account, despite the p-values of the model comparisons were smaller than the .05 level, we concluded that the small ΔCFA (all ≥ -.010) and ΔRMSEA values (all ≤ .015) supported the invariance hypothesis (Chen, 2007). Therefore, the measures of CPS task were strictly invariant across cohort groups.

Reference

Chen, F. F. (2007). Sensitivity of goodness of fit indexes to lack of measurement invariance. *Structural Equation Modeling, 14*(3), 464–504. doi:10.1080/10705510701301834.

Greiff, S., Wüstenberg, S., & Funke, J. (2012). Dynamic Problem Solving: A new measurement perspective. Applied Psychological Measurement, 36(3), 189–213. doi:10.1177/0146621612439620.

Jarušek, P. & Pánek, R. (2010). *Human problem solving: Sokoban case study*. Technical Report FIMU-RS-2010-01, Masaryk University Brno.

Li, J., Zhang, B., Du, H., Zhu, Z., & Li, Y. M. (2015). Metacognitive planning: Development and validation of an online measure. *Psychological Assessment, 27(1),* 260-271. doi:10.1037/pas0000019.

Muthén, L.K. & Muthén, B.O. (2012). *Mplus User's Guide. Seventh Edition*. Los Angeles, CA: Muthén & Muthén.

Schweizer, F., Wüstenberg, S., & Greiff, S. (2013). Validity of the MicroDYN approach: Complex problem solving predicts school grades beyond working memory capacity. *Learning and Individual Differences, 24*, 42–52. doi:10.1016/j.lindif.2012.12.011.
